# Prediction of System Parameters of Carbon-Based Composite Structure for Different Carbon Fiber Orientations with Mode Information at Reference Angle Only

**DOI:** 10.3390/ma14247626

**Published:** 2021-12-11

**Authors:** Chan-Jung Kim

**Affiliations:** Department of Mechanical Design Engineering, Pukyong National University, Busan 48513, Korea; cjkim@pknu.ac.kr; Tel.: +82-51-629-6169

**Keywords:** system parameter prediction, viscous damping coefficient, resonance frequency, system parameter ratio, carbon fiber orientation

## Abstract

The prediction of system parameters is important for understanding the dynamic behavior of composite structures or selecting the configuration of laminated carbon in carbon-based composite (CBC) structures. The dynamic nature of CBC structures allows the representation of system parameters as modal parameters in the frequency domain, where all modal parameters depend on the carbon fiber orientations. In this study, the variation in the system parameters of a carbon fiber was derived from equivalent modal parameters, and the system parameters at a certain carbon fiber orientation were predicted using the modal information at the reference carbon fiber orientation only and a representative curve-fitted function. The target CBC structure was selected as a simple rectangular structure with five different carbon fiber orientations, and the modal parameters were formulated based on a previous study for all modes. Second-order curve-fitted polynomial functions were derived for all possible cases, and representative curve-fitting functions were derived by averaging the polynomial coefficients. The two system parameters were successfully predicted using the representative curve-fitting function and the modal information at only the reference carbon fiber orientation, and the feasibility of parameter prediction was discussed based on an analysis of the error between the measured and predicted parameters.

## 1. Introduction

Carbon-based composite (CBC) materials are widely applied in various industries owing to their high strength-to-weight ratio as well as the development of mass producibility [1,2,3]. Several applications of carbon-fiber-reinforced composite materials have been reported in previous studies. In civil engineering, the axial behavior of long circular concrete-filled fiber-reinforced polymer tube columns [4], strengthening methods for damaged high-temperature steel pipelines [5], and hybrid slabs manufactured using CBC and concrete [6] were investigated based on reinforcements with carbon-fiber composites. In automotive engineering, the topology optimization algorithm was applied to a battery-hanging structure with laminated CBC material [7], and a center pillar component with composite reinforcements was developed using a hybrid molding method [8]. In addition, embedded sensing characteristics were obtained by embedding hybridized carbon nanomaterials into CBC composites [9]. Manufacturing problems pertaining to CBC structures are primary issues that have hindered their wide development in industry, and many studies have been conducted to address these issues. The single-point cutting fracture behavior of pre-impregnated CBC sheets was investigated with respect to the effect of fiber angle [10], and the effects of varying the drilling feed and speed conditions on fiber pull-out geometries and hole quality parameters were investigated [11]. In addition, the clinching process between aluminum alloy and CBC sheets was investigated [12], and its applicability to the micro-drilling of multidirectional CBC structures was verified experimentally [13]. Because the anisotropic characteristics of CBC materials demonstrate vastly different mechanical properties compared with conventional isotropic materials (i.e., carbon steel and aluminum alloy), extensive studies have been conducted to identify the characteristics of CBC materials. The prediction of fatigue life until crack initiation in CBC structures was investigated [14], and beam structures retrofitted with reinforcements were compared with respect to the resultant structural stiffness [15]. In addition, the bonding characteristics of CBC specimens [16], surface treatment to enhance the mechanical properties of CBC structures [17], and mechanical bearing strength and failure modes of composite-to-metal jointing structures [18] have been investigated in previous studies. These mechanical properties were associated with the static properties, and the dynamic mechanical properties were investigated as well. The effect of laminated ethylene vinyl acetate sheets on CBC laminates was experimentally proven to enhance the impact absorption energy [19]. The dynamic characteristics of CBC structures can be identified using the well-known modal test technique [20,21]. As such, most system problems regarding the dynamic behavior of CBC structures can be solved using the identified modal information from modal tests in previous studies [22,23,24,25]. Because the dynamic behavior of the CBC structure changes according to the orientation of the carbon fibers in the laminated structure, the identified modal parameters are highly sensitive to the carbon fiber orientation. The frequency response function or modal parameters of the CBC structure were found to be dependent on three parameters: temperature, spectral loading pattern, and carbon fiber orientation [26,27,28]. The mode shape of the clamped CBC structure was compared with the modal assurance criterion (MAC) after conducting an experimental modal test, and the variation in the system parameters, the resonance frequency, and the corresponding mode shape were evaluated in four modes of interest [29]. In recent studies, a mode order tracking method was developed for different carbon fiber orientations with multiple indicators, i.e., the MAC value, resonance frequency, and modal damping ratio [30], and the relationship between the structural stiffness and viscous damping coefficient was found to be proportional for all modes of interest [31]. In this study, the system parameters, the resonance frequency and the viscous damping coefficient, were predicted using system parameter information at the reference carbon fiber orientation only. The system parameters can be predicted only if the representative curve-fitted function is pre-determined from a separate modal test process at the target CBC structure. The feasibility of the prediction of system parameters was determined using modal information from previous studies [30,31], and the second-order curve-fitting functions were calculated from the pre-determined dataset. The accuracy of the predicted system parameters was evaluated through an analysis of the error between the measured and predicted parameters. Finally, the importance of the predicted system parameters is discussed with respect to the design guidelines for the selection of the carbon fiber orientation in the CBC structure. 

## 2. Theoretical Background for System Parameter Prediction

Mechanical systems can be modeled with mechanical components, i.e., mass, damper, and spring, if they are a linear system [20,21]. The CBC system comprises two main components: a carbon fiber and a polymer matrix. As such, a one-degree-of-freedom (DOF) CBC structure can be expressed, as shown in Equation (1) [20,21,31].

(1) $$m\ddot{x}\left(t\right)+c_{eq}\dot{x}\left(t\right)+k_{eq}x\left(t\right)=0$$

Here, m is the mass, and $c_{eq}$ and $k_{eq}$ are the equivalent damping coefficient and spring coefficient, respectively. The two equivalent system parameters can be expressed by the linear combination of the two main components of the CBC structure, i.e., the carbon fiber and polymer matrix, as formulated in Equations (2) and (3) [21,31].

(2) $$c_{eq}={\left(\frac{1}{c_{C}}+\frac{1}{c_{M}}\right)}^{-1}$$

(3) $$k_{eq}=k_{C}+k_{M}$$

Here, $c_{C}$ and $c_{M}$ are the damping coefficients of the carbon fiber and polymer matrix, respectively; $k_{C}$ and $k_{M}$ are the spring coefficients of the carbon fiber and polymer matrix, respectively. Equation (1) can be transformed into a modal coordinate by normalizing the system parameters (${\bar{c}}_{eq}$, ${\bar{k}}_{eq}$) with m, and the normalized system parameters can be expressed using modal parameters. The normalized system parameters are shown in Equations (4) and (5), and the transformed governing equations are expressed in Equation (6).

(4) $$\frac{c_{eq}}{m}={\left(\frac{1}{2\xi_{C}\omega_{n,C}}+\frac{1}{2\xi_{M}\omega_{n,M}}\right)}^{-1}$$

(5) $$\frac{k_{eq}}{m}={\left(\omega_{n,C}\right)}^{2}+{\left(\omega_{n,M}\right)}^{2}$$

(6) $$\ddot{x}\left(t\right)+{\left(\frac{1}{2\xi_{C}\omega_{n,C}}+\frac{1}{2\xi_{M}\omega_{n,M}}\right)}^{-1}\dot{x}\left(t\right)+\left({\left(\omega_{n,C}\right)}^{2}+{\left(\omega_{n,M}\right)}^{2}\right)x\left(t\right)=0$$

Here, $\xi_{C}$ and $\xi_{M}$ are the modal damping ratios of the carbon fiber and polymer matrix, respectively; $\omega_{n,C}$ and $\omega_{n,M}$ are the resonance frequencies of the carbon fiber and polymer matrix, respectively. The CBC structure should be extended into a multi-DOF system in practice because the dynamic characteristics of the CBC structure can be representative of the multi-DOF model instead of the one-DOF model in Equation (6). If the CBC system is assumed to be an N-DOF system, then an appropriate model can be formulated using Equation (7) under the similar expression shown in Equation (6). In addition, the normalized viscous damping coefficient in the *i*th mode (${\bar{c}}_{eq,i}$) and the normalized stiffness coefficient (${\bar{k}}_{eq,i}$) in the *i*th mode are expressed in Equations (8) and (9), respectively.

(7) $$\begin{array}{l} \left[\begin{array}{ccc} 1 & & zeros\\ & \ddots & \\ zeros & & 1 \end{array}\right]\ddot{R}\\ +\left[\begin{array}{ccc} {\left(\frac{1}{2\xi_{1,C}\omega_{n_{1},C}}+\frac{1}{2\xi_{1,C}\omega_{n_{1},M}}\right)}^{-1} & & zeros\\ & \ddots & \\ zeros & & {\left(\frac{1}{2\xi_{N,C}\omega_{n_{1},C}}+\frac{1}{2\xi_{N,C}\omega_{n_{1},M}}\right)}^{-1} \end{array}\right]\dot{R}\\ +\left[\begin{array}{ccc} {\left(\omega_{n_{1},C}\right)}^{2}+{\left(\omega_{n_{1},M}\right)}^{2} & & zeros\\ & \ddots & \\ zeros & & {\left(\omega_{n_{N},C}\right)}^{2}+{\left(\omega_{n_{N},M}\right)}^{2} \end{array}\right]R=\left[\begin{array}{c} 0\\ \vdots \\ 0 \end{array}\right] \end{array}$$

(8) $${\bar{c}}_{eq,i}=2\xi_{i}\omega_{n_{i}}={\left(\frac{1}{2\xi_{i,C}\omega_{n_{i},C}}+\frac{1}{2\xi_{i,M}\omega_{n_{i},M}}\right)}^{-1}$$

(9) $${\bar{k}}_{eq,i}={\left(\omega_{n_{i}}\right)}^{2}={\left(\omega_{n_{i},C}\right)}^{2}+{\left(\omega_{n_{i},M}\right)}^{2}$$

Here, $R={\left[\begin{array}{ccc} x_{1}\left(t\right) & x_{2}\left(t\right) & \cdots \end{array}\;\begin{array}{cc} x_{N-1}\left(t\right) & x_{N}\left(t\right) \end{array}\right]}^{T}$ is the column vector that represents the response of the CBC structure. In addition, $\xi_{i,C}$ and $\xi_{i,M}$ are the modal damping ratios of the carbon fiber and polymer matrix at the *i*th mode, respectively; $\omega_{n_{i},C}$ and $\omega_{n_{i},M}$ are the resonance frequencies of the carbon fiber and polymer matrix at the *i*th mode, respectively. 

The characteristics of the CBC structure are associated closely with the carbon fiber orientation, and the system parameters shown in Equations (8) and (9) should be addressed by considering the anisotropic mechanical properties. If the carbon fibers in the CBC structure are assumed to be aligned at a certain angle ($\theta_{j}$), then the system parameters are functions of the carbon fiber orientation, expressed as follows.

(10) $${\bar{c}}_{eq,i}\left(\theta_{j}\right)={\left(\frac{1}{2\xi_{i,C}\left(\theta_{j}\right)\omega_{n_{i},C}\left(\theta_{j}\right)}+\frac{1}{2\xi_{i,M}\left(\theta_{j}\right)\omega_{n_{i},M}\left(\theta_{j}\right)}\right)}^{-1}$$

(11) $${\bar{k}}_{eq,i}\left(\theta_{j}\right)={\left(\omega_{n_{i},C}\left(\theta_{j}\right)\right)}^{2}+{\left(\omega_{n_{i},M}\left(\theta_{j}\right)\right)}^{2}$$

The system parameters in Equations (10) and (11) can be formulated using a combination of coefficients for both the carbon fiber and polymer matrix. In a published paper [31], the sensitivity of each system parameter at a certain angle $\theta_{j}$ is expressed as shown in Equations (12) and (13), with respect to the reference carbon fiber orientation, $\theta_{1}$.

(12) $$\frac{{\bar{c}}_{C,i}\left(\theta_{j}\right)}{{\bar{c}}_{C,i}\left(\theta_{1}\right)}\approx \frac{{\bar{c}}_{eq,i}\left(\theta_{j}\right)}{{\bar{c}}_{eq,i}\left(\theta_{1}\right)}$$

(13) $$\frac{{\bar{k}}_{C,i}\left(\theta_{j}\right)}{{\bar{k}}_{C,i}\left(\theta_{1}\right)}=1-\frac{{\bar{k}}_{eq,i}\left(\theta_{1}\right)-{\bar{k}}_{eq,i}\left(\theta_{j}\right)}{{\bar{k}}_{eq,i}\left(\theta_{1}\right)-{\bar{k}}_{eq,i}\left(\theta_{*}\right)}$$

Here, ${\bar{k}}_{eq,i}\left(\theta_{*}\right)$ is the lowest value of the stiffness coefficient among the candidate carbon fiber orientations in the *i*th mode. 

The equivalent system parameters can be predicted using Equations (12) and (13). If the system parameters at $\theta_{1}$ as well as the ratio of each parameter (c¯C,i(θj)c¯C,i(θ1) and k¯C,i(θj)k¯C,i(θ1)) in the left term can be identified, then the equivalent modal parameters of the CBC structure can be predicted at a certain angle of the carbon fiber, $\theta_{j}$. Meanwhile, the viscous damping coefficient ${\bar{c}}_{eq,i}\left(\theta_{j}\right)$ can be predicted using the ratio of the viscous damping ratio of the carbon fiber and ${\bar{c}}_{eq,i}\left(\theta_{1}\right)$. The prediction of ${\bar{k}}_{eq,i}\left(\theta_{j}\right)$ is not as simple as that of ${\bar{c}}_{eq,i}\left(\theta_{j}\right)$ because it requires two equivalent stiffness coefficients, ${\bar{k}}_{eq,i}\left(\theta_{1}\right)$ and ${\bar{k}}_{eq,i}\left(\theta_{*}\right)$, as well as the stiffness ratio of the carbon fiber to be obtained. Therefore, the case involving the stiffness coefficient was reviewed based on Equation (13), which was recalled from a previous study [31], as follows.

(14) $$\frac{{\bar{k}}_{C,i}\left(\theta_{j}\right)}{{\bar{k}}_{C,i}\left(\theta_{1}\right)}=1-\frac{{\bar{k}}_{eq,i}\left(\theta_{1}\right)-{\bar{k}}_{eq,i}\left(\theta_{j}\right)}{{\bar{k}}_{C,i}\left(\theta_{1}\right)}$$

The equivalent stiffness coefficient is a series combination of the stiffness coefficients of the carbon fiber and polymer matrix (see Equation (5)), and it can be assumed that the stiffness coefficient is maximum at the reference angle ($\theta_{1}$). As discussed in [31], the stiffness coefficient of a polymer matrix is not affected by the carbon fiber orientation, and the stiffness coefficient of the polymer matrix is small compared with that of the carbon fiber. Therefore, ${\bar{k}}_{C,i}\left(\theta_{1}\right)$ can be replaced by ${\bar{k}}_{eq,i}\left(\theta_{1}\right)$ under the minimum error owing to the maximum stiffness condition at $\theta_{1}$, and Equation (14) can be reformulated as follows.

(15) $$\frac{{\bar{k}}_{C,i}\left(\theta_{j}\right)}{{\bar{k}}_{C,i}\left(\theta_{1}\right)}\approx 1-\frac{{\bar{k}}_{eq,i}\left(\theta_{1}\right)-{\bar{k}}_{eq,i}\left(\theta_{j}\right)}{{\bar{k}}_{eq,i}\left(\theta_{1}\right)}=\frac{{\bar{k}}_{eq,i}\left(\theta_{j}\right)}{{\bar{k}}_{eq,i}\left(\theta_{1}\right)}$$

The increase or decrease in the equivalent system parameters are not always started from the reference angle $\theta_{1}$, therefore, another carbon fiber orientation $\phi_{1}$ is introduced as the starting point of the variation of the system parameters. The variation of the equivalent system parameters can be efficiently identified by the rearrangement of the system parameters according to the increase of $\phi_{j}$ which is the *j*th increased carbon fiber orientation from $\phi_{1}$. If one system parameter is increased or decreased from the carbon fiber orientation $\phi_{1}$, then the other system parameter will increase or decrease, respectively, because the relationship between the two system parameters is proportional [31]. The ratio of the viscous damping coefficient and stiffness coefficient at the *i*th mode, as shown in Equations (12) and (15), respectively, can be approximately expressed by the curve-fitted function from all the carbon fiber orientation sets and the estimated fitting curves were defined as $\Gamma_{\bar{c},i}$ and $\Gamma_{\bar{k},i}$ (see Equations (16) and (17)), respectively.

(16) $$\Gamma_{\bar{c},i}\left(\phi_{j}\right)\approx \frac{{\bar{c}}_{C,i}\left(\phi_{j}\right)}{{\bar{c}}_{C,i}\left(\phi_{1}\right)}$$

(17) $$\Gamma_{\bar{k},i}\left(\phi_{j}\right)\approx \frac{{\bar{k}}_{C,i}\left(\phi_{j}\right)}{{\bar{k}}_{C,i}\left(\phi_{1}\right)}$$

As shown in Equations (12) and (15), the equivalent system parameters, the viscous damping coefficient, and stiffness coefficient of the CBC structure can be directly predicted from the curve-fitting functions $\Gamma_{\bar{c},i}$ and $\Gamma_{\bar{k},i}$. However, the main focus is to estimate the system parameters at certain carbon fiber orientations based on prior knowledge regarding the measured data at a reference carbon fiber only, such that the system parameters of the CBC structure can be predicted without requiring any further measured datasets. One of the solutions for predicting the system parameters is to introduce a representative curve-fitting function if the representative function can encompass all modes of interest in the CBC structure. It is assumed that the two representative functions, defined as $\Gamma_{\bar{c}}$ and $\Gamma_{\bar{k}}$, are applicable to the prediction of system parameters, the viscous damping coefficient and the stiffness coefficient. Therefore, the approximated system parameters in a certain carbon fiber orientation ($\phi_{j}$) can be expressed as shown in Equations (18) and (19).

(18) $${\bar{c}}_{eq,i}\left(\phi_{j}\right)={\bar{c}}_{eq,i}\left(\phi_{1}\right)\Gamma_{\bar{c},i}\left(\phi_{j}\right)\approx {\bar{c}}_{eq,i}\left(\phi_{1}\right)\Gamma_{\bar{c}}\left(\phi_{j}\right)$$

(19) $${\bar{k}}_{eq,i}\left(\phi_{j}\right)={\bar{k}}_{eq,i}\left(\phi_{1}\right)\Gamma_{\bar{k},i}\left(\phi_{j}\right)\approx {\bar{k}}_{eq,i}\left(\phi_{1}\right)\Gamma_{\bar{k}}\left(\phi_{j}\right)$$

## 3. Identification of Modal Parameters of CBC Specimens

**Figure 1:**
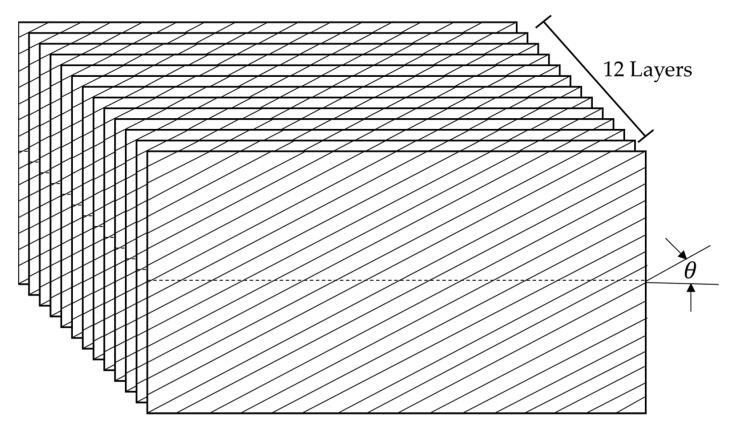
Carbon fiber orientation in multilayered CBC specimen [31].

CBC specimens were prepared based on five different angles of the carbon fiber, namely, $\theta_{1}=0{}^{\circ}$, $\theta_{2}=30{}^{\circ}$, $\theta_{3}=45{}^{\circ}$, $\theta_{4}=0{}^{\circ}$, and $\theta_{5}=90{}^{\circ}$, and the configuration of the simple structure was 80 mm (W) × 150 mm (L) × 3 mm (H), as illustrated in Figure 1. The CBC specimens were prepared using a large composite plate, which was manufactured using a 12-layered pre-impregnated material (USN 250A, SK Chemical, Seongnam, Korea). The material USN 250A was composed of T700 carbon fiber (12k, Toray, Tokyo, Japan) and a polymer matrix (epoxy resin); it was cured under an autoclave process (at 125 °C maximum) [32]. 

**Figure 2:**
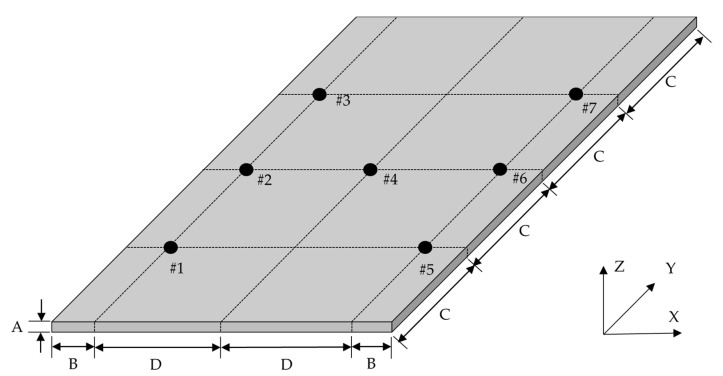
Configuration of CBC specimen and sensor attachment locations: A: 3 mm, B: 10 mm, C: 37.5 mm, D: 30 mm [31].

The modal parameters of the CBC specimens were identified using the experimental impact test technique. The frequency response functions (FRFs) between the impact point and response locations were obtained using an impact hammer (Model: 5800B3, Dytran, Chatsworth, CA, USA) and uniaxial accelerometers (Model: 3225F2, Dytran). The impact force was assigned at #4, and the response accelerations were measured at #1–#7, as shown in Figure 2. Because the total mass of the CBC specimen was 56.5 (g), the mass loading effect from the accelerometers is negligible owing to the small total mass of the accelerometers (1 (g) × 7= 7 (g)). The sensor locations of the CBC specimens were selected to provide sufficient space between them. 

**Figure 3:**
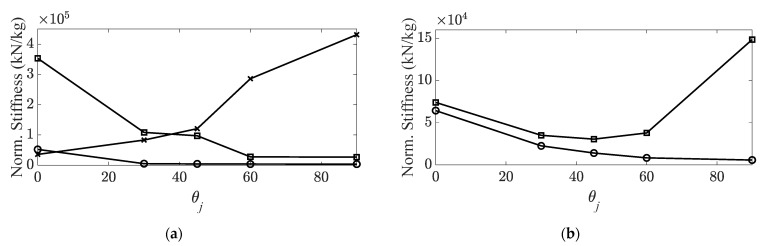
Variations in structural stiffness with respect to carbon fiber orientation. (**a**) Bending modes, 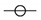: first bending mode; 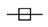: second bending mode; 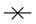: third bending mode; (**b**) torsional modes, 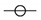: first torsional mode; 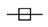: second torsional mode [30,31].

**Figure 4:**
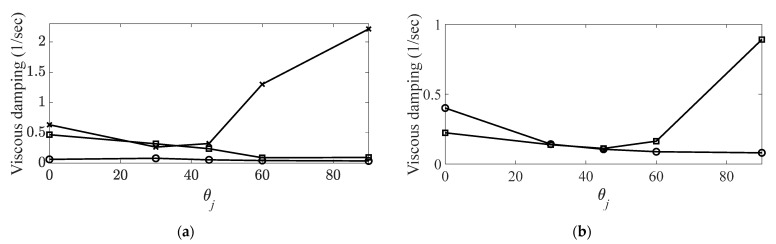
Variations in viscous damping coefficient with respect to carbon fiber orientation. (**a**) Bending modes, 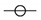: first bending mode; 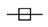: second bending mode; 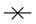: third mode.; (**b**) torsional modes, 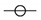: first torsional mode; 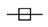: second torsional mode [30,31].

A frequency range between 10 (Hz) and 4096 (Hz) was selected, and ten times the average of the measured FRFs was used to eliminate noise in the FRFs of the CBC specimens. The constraint condition of the CBC specimens was set as free–free with rubber bands, and all data were recorded using an eight-channel data-acquisition device (Test.Lab/Siemens/Germany) [30,31]. The system parameters of the CBC specimens were calculated using the PolyMax algorithm from the Test.Lab equipment. Because the resonance frequency and the corresponding eigenvector were changed based on the carbon fiber orientation, the modes of interest were tracked using an MAC, as well as the variations in the resonance frequency and viscous damping coefficient reported previously [30]. MAC values were calculated using MATLAB software (MathWorks, Natick, MA, USA). The five tracked modes of interest of the CBC structure are summarized in Table 1, and the variation of each system parameter is illustrated in Figure 3 and Figure 4. 

## 4. Prediction of Modal Parameters

**Figure 5:**
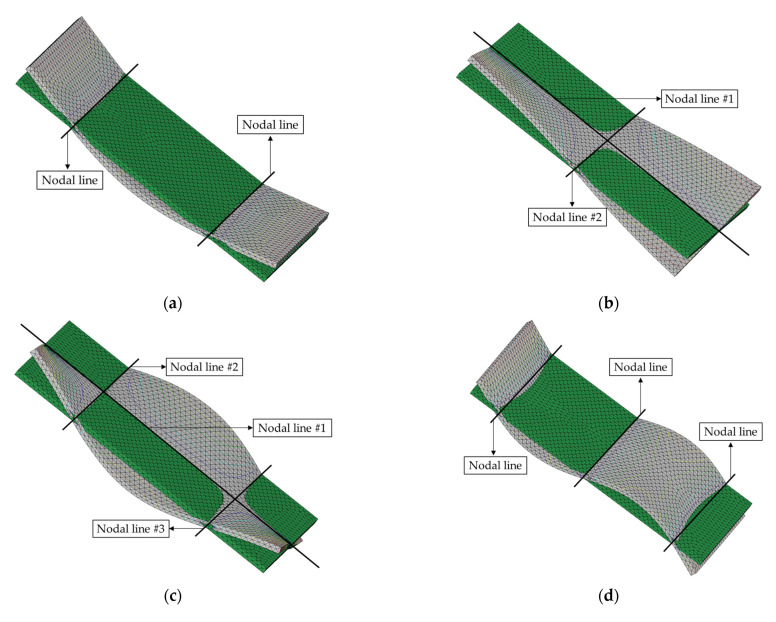

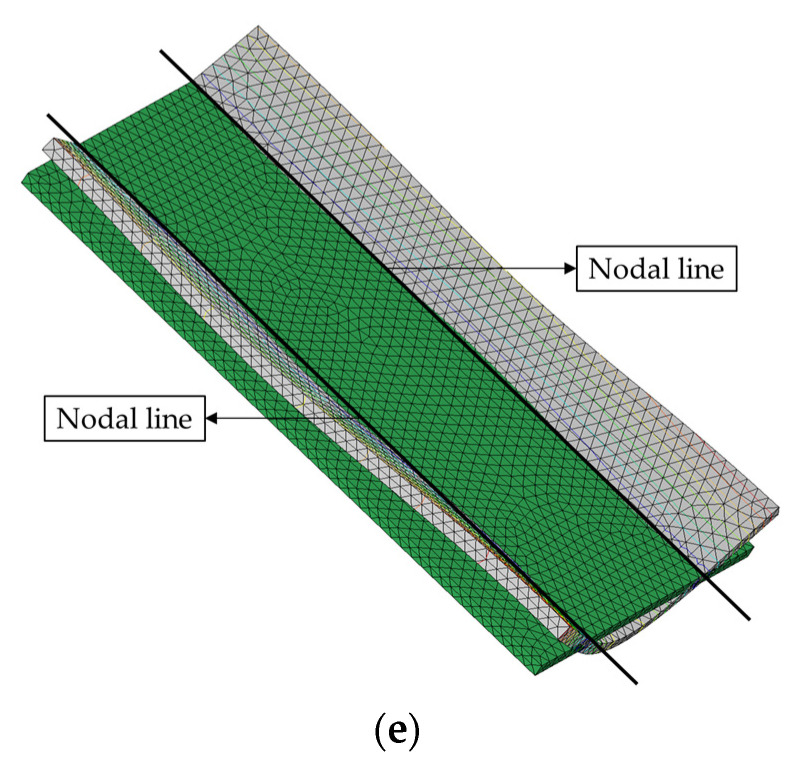
Configuration of nodal lines in each mode shapes. Undeformed mode shapes (green color) overlapped with deformed mode shape (gray color): (**a**) first mode (first bending); (**b**) second mode (first torsional); (**c**) third mode (second bending); (**d**) fourth mode (second bending); (**e**) fourth mode (third bending).

The system parameters were first predicted by selecting $\phi_{1}$ in each mode of the CBC structure. Generally, the nodal line can be observed from the calculated mode shape from the measured FRFs; however, this method is not appropriate for this study because the limited number of measurement points were insufficient to represent the nodal lines (see Figure 2) in the mode shapes. Hence, the mode shapes analyzed in a previous study were used to identify the nodal lines in each mode of interest [30,31], as illustrated in Figure 5. Here, the theoretical modal analysis had been conducted using Virtual.Lab software (Siemens, Munich, Germany). 

The nodal lines showed the same direction in all three bending modes; however, two directional orthogonal nodal lines were observed in two torsional modes, as shown in Figure 5. For all bending modes, the structural stiffness was maximized when the carbon fiber orientation was orthogonal to the direction of the nodal lines. Therefore, the maximum stiffness cases ($\emptyset_{1}$) can be expected at $\theta_{1}$, $\theta_{1}$, and $\theta_{5}$ for the first, second, and third bending modes, respectively. However, the maximum structural stiffness condition was complex for the two torsional modes. For the first torsional mode, the two nodal lines (#1 and #2) were crossed at the center of the CBC specimen in an orthogonal direction. The structural torsional stiffness was much higher for the direction of nodal line #2 as compared with that of nodal line #1 owing to the different lengths of the CBC specimen. The effect of the reinforced structural torsional stiffness can be expected to be maximum at $\theta_{1}$. This implies that the reinforced direction at $\theta_{1}$ and nodal line #2 were orthogonal to carbon fiber orientation $\theta_{1}$. In the second torsional mode, nodal line #1 was crossed by two parallel lines (#2 and #3) at two different points. Therefore, it was difficult to predict the direction of the reinforcement from the carbon fiber that affected the second torsional mode. The experimental results shown in Figure 3 indirectly indicate that the maximum torsional stiffness condition was $\theta_{1}$ or $\theta_{5}$ for the second torsional mode. The maximum stiffness case is summarized in Table 2 based on the nodal lines in each mode. 

**Figure 6:**
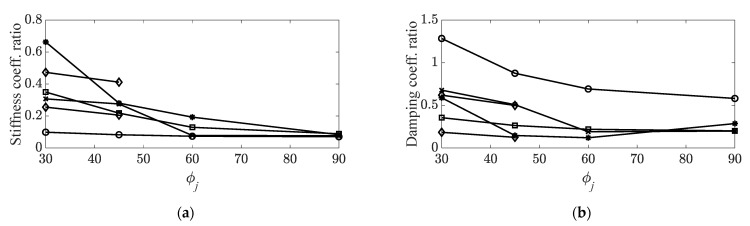
Rearranged variations in measured data. (**a**) Stiffness coefficient ratios, 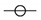: first bending mode; 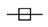: first torsional mode; 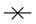: second bending mode; 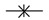: third bending mode; 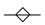: second torsional mode; (**b**) damping coefficient ratios, 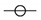: first bending mode; 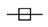: first torsional mode; 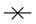: second bending mode; 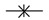: third bending mode, 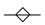: second torsional mode.

Using the selected angle sequence for predicting the ratio of the system parameters, viscous damping coefficient, and stiffness coefficient, the rearranged variation of the ratios can be illustrated as shown in Figure 6. In the second torsional mode, the system parameters began to vary at two different $\phi_{1}$ points owing to the coexistence of two maximum parameter cases ($\theta_{1}$ and $\theta_{5}$). 

**Figure 7:**
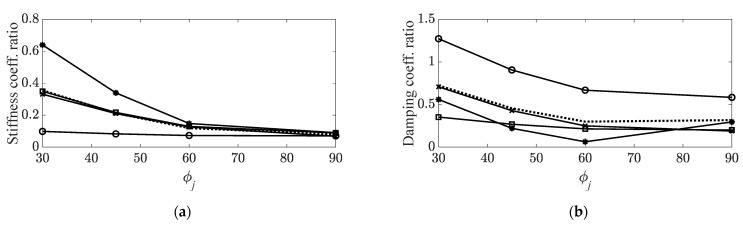
Curve-fitted variations in system parameters: (**a**) Stiffness coefficient ratios, 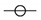: first bending mode; 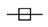: first torsional mode; 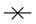: second bending mode; 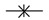: third bending mode; 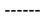: averaged curve; (**b**) damping coefficient ratios, 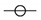: first bending mode; 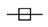: first torsional mode; 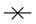: second bending mode; 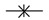: third bending mode; 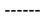: averaged curve.

The curve-fitted functions, $\Gamma_{\bar{c},i}$ and $\Gamma_{\bar{k},i}$, obtained from the measured data in Figure 6, can be represented as second-order polynomial functions using MATLAB software (MathWorks, Natick, MA, USA), as illustrated in Figure 7. In addition, the representative fitted functions for the two system parameters were calculated by averaging the coefficients of the second-order polynomial functions, as illustrated in the figure below. Curve fitting from the dataset in the second torsional mode was not conducted because the second-order curve-fitted function required at least three datasets, but the length of the current dataset was two. 

**Figure 8:**
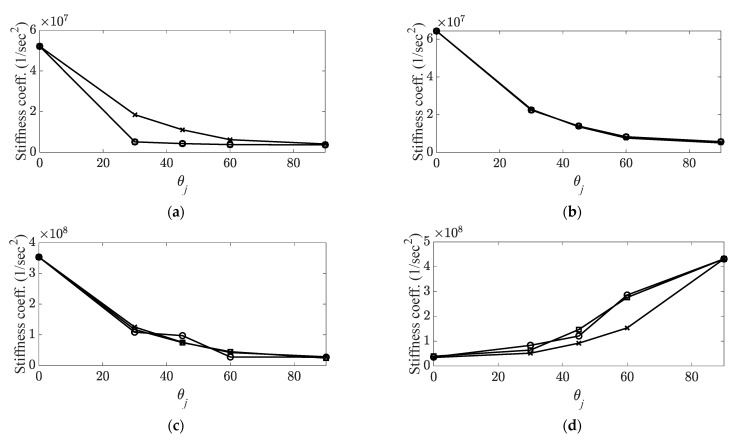
Prediction of stiffness coefficient. 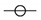: measured parameter; 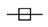: predicted parameter with direct fitting curve function; 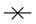: predicted parameter with representative fitting-curve function. (**a**) First mode (first bending); (**b**) second mode (first torsional); (**c**) fourth mode (second bending); (**d**) fifth mode (third bending).

**Figure 9:**
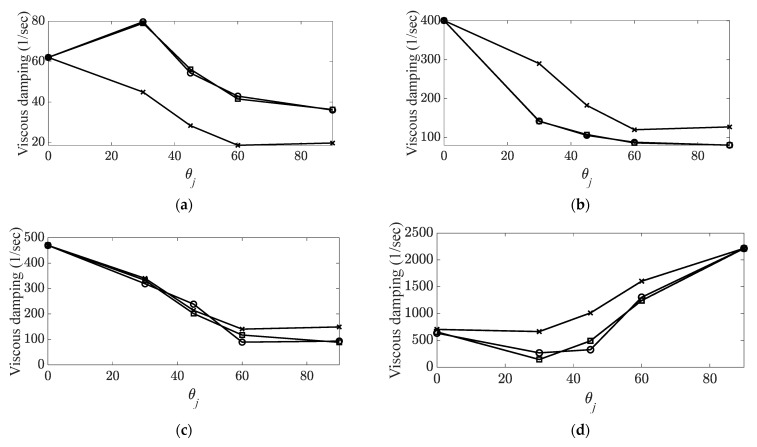
Prediction of viscous damping coefficient. 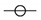: measured parameter; 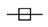: predicted parameter with direct fitting curve function; 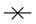: predicted parameter with representative fitting-curve function. (**a**) First mode (first bending); (**b**) second mode (first torsional); (**c**) fourth mode (second bending); (**d**) fifth mode (third bending).

The system parameters were predicted directly using the corresponding curve-fitted polynomial functions ($\Gamma_{\bar{c},i}$ and $\Gamma_{\bar{k},i}$). For the case involving the representative curve-fitting functions, $\Gamma_{\bar{c}}$ and $\Gamma_{\bar{k}}$, the indirect prediction results were calculated to compare them with the results estimated directly from each curve-fitted polynomial function. The representative curve-fitting polynomial functions are summarized in Table 3, and the prediction of each system parameter is shown in Figure 8 and Figure 9. The two-representative curve-fitting in Table 3 shows similar polynomial functions, which implies that the variations in the two system parameters were similar as the carbon fiber orientation increased. In a previous study [31], the relationship between the two system parameters was proven to be proportional to the increase in the carbon fiber orientation; therefore, the similar results shown in Table 3 are reasonable. 

The prediction of each system parameter showed that the direct prediction from the curved-fitting function from each measured data was more accurate than the indirect prediction from the representative curve-fitting function shown in Table 3. Error between the measured parameter and two different predicted parameters was analyzed, for which the parameters were derived using the direct curve-fitted function and the representative function, as shown in Table 4. Here, the relative error denotes the absolute error divided by the measured error. 

The reliable results obtained from the direct prediction depended on the performance of the applied curve-fitting algorithm; hence, the small error values (8.11% for the viscous damping coefficient and 8.74% for the stiffness coefficient) did not significantly affect the prediction of the system parameters. In other words, if all measured parameter sets for the possible carbon fiber orientations are well prepared in the CBC structure, then the prediction of the system parameters can be regarded as redundant. However, the system parameters calculated from the representative fitted function should be considered when predicting the system parameters at any carbon fiber orientation. The representative functions (see Table 3) are the averaged functions from the previous activity, and these functions can be assumed as pre-knowledge information. The two system parameters decreased in a similar trend for all modes of interest; therefore, the derived representative functions were applicable for all carbon angles. However, the error from the representative fitting function indicated relatively high values (36.20% for the viscous damping coefficient and 49.87% for the stiffness coefficient) as compared with the direct prediction case. Therefore, the indirectly predicted values were used to identify the variation in the system parameters with the increase in the carbon fiber orientation from the reference value. 

If the representative fitted functions ($\Gamma_{\bar{c}}$ and $\Gamma_{\bar{k}}$) can be prepared for the CBC structure, then the two system parameters can be predicted using the system parameters at a reference carbon fiber orientation, as well as the representative functions shown in Equations (18) and (19), respectively. The two system parameters are directly matched with the modal parameters at all modes of interest, and the dynamic behavior of the CBC structure can be expressed appropriately in the frequency domain expressed in Equation (7). Therefore, the proposed prediction method enables system engineers of CBC structures to determine the system parameters (i.e., the resonance frequency and viscous damping coefficient) at a certain carbon fiber orientation virtually before manufacturing the structures. 

The limitations of the proposed method can be briefly discussed from a practical perspective. The first is the possibility of a representative curved-fitting function for the system parameters. The representative curve-fitted function is valid owing to a similar variation trend in the system parameters for the five modes of interest. If the mode number is extended to a higher frequency range, then a similar variation in the system parameters cannot be guaranteed. If the variation in the system parameter shows a completely different trend at higher modes, then the simple averaged approximated functions cannot be represented at the appropriate resonance frequency. The second limitation is that the representative function depends significantly on the configuration of the CBC structure. In practice, the CBC structure may be designed using a configuration other from the current simple rectangular shape such that the resonance frequencies and the corresponding mode shapes may be altered from the current results. Subsequently, the selection of $\phi_{1}$ may change depending on the calculated mode shape. In particular, the prediction of the system parameters should be valid for the general cases, such as the complex shapes and different boundary conditions, to demonstrate the novelty of the proposed prediction method. This implies that the proposed method was focused on the feasibility of the prediction of system parameters in the CBC structure over the different carbon fiber orientations under the measured data at a reference angle only. The application of the proposed method for the complex shape or boundary conditions of the CBC structure is being considered for future studies. 

## 5. Conclusions

The prediction of the system parameters for a CBC structure was formulated using curve-fitted polynomial functions as well as modal information at the reference carbon fiber orientation only. For the prepared simple rectangular CBC structure with five different carbon fiber orientations, all modal parameters were taken from previous reports, and the variation in the system parameters was expressed using second-order curve-fitted polynomial functions. Because the two system parameters, namely, the structural stiffness and the modal damping coefficient, were rearranged based on the parameter value order in each mode, the variation trend for all modes of interest decreased as the rearranged carbon fiber orientation $\phi_{j}$ increased. In addition, the two-representative curve-fitted functions indicated similar coefficients because of the linear relationship between the viscous damping coefficient and the stiffness coefficient from the previous study. The indirect prediction of system parameters from the representative curve-fitted function indicated a relatively low accuracy (36.20% for the viscous damping coefficient, and 49.87% for the stiffness coefficient) as compared with the direct prediction from the curve-fitted function with each measured dataset. However, the system parameter prediction appeared to be compatible with the representative curve-fitting function because the variation trend was not affected by the increase in the carbon fiber orientation. If a representative variation curve can be obtained for the target CBC structure, then the expected system parameters can be estimated for any carbon fiber orientation. Therefore, the proposed system parameter prediction procedure can help provide baselines for the configuration of carbon fiber orientations. 

## Figures and Tables

**Table 1:** Measured modal parameters of CBC specimen for five different carbon fiber orientations [30].

Specimen	Resonance Frequency (Hz)	Modal Damping Ratio (%)	Mode Shape
CBC specimen #1 $\left(\theta_{1}=0{}^{\circ}\right)$	1149.1	0.4	Bending (first)
	1276.1	2.5	Torsional (first)
	1368.7	1.3	Torsional (second)
	2990.9	1.3	Bending (second)
	951.0	5.3	Bending (third)
CBC specimen #2 $\left(\theta_{2}=30{}^{\circ}\right)$	360.6	0.39	Bending (first)
	754.5	0.21	Torsional (first)
	941.1	0.82	Torsional (second)
	1657.6	0.01	Bending (second)
	1450.4	0.55	Bending (third)
CBC specimen #3 $\left(\theta_{3}=45{}^{\circ}\right)$	330.4	1.3	Bending (first)
	595.6	1.4	Torsional (first)
	878.0	1.0	Torsional (second)
	1568.9	1.2	Bending (second)
	1749.2	1.5	Bending (third)
CBC specimen #4 $\left(\theta_{4}=60{}^{\circ}\right)$	310.6	1.1	Bending (first)
	458.5	1.5	Torsional (first)
	979.0	1.3	Torsional (second)
	835.0	0.9	Bending (second)
	2690.4	3.9	Bending (third)
CBC specimen #5 $\left(\theta_{5}=90{}^{\circ}\right)$	305.1	0.9	Bending (first)
	380.0	1.7	Torsional (first)
	1938.5	3.7	Torsional (second)
	824.1	0.9	Bending (second)
	3305.1	5.3	Bending (third)

**Table 2:** Maximum parameter case and corresponding angle sequence for prediction.

Mode	Maximum Parameter Case	Rearranged Angle Sequence	Corresponding Angle Increase
First bending	$\theta_{1}$	$\theta_{1}$ $⟶\theta_{2}$ $⟶\theta_{3}$ $⟶\theta_{4}$ $⟶\theta_{5}$	$\phi_{1}$ $⟶\phi_{2}$ $⟶\phi_{3}$ $⟶\phi_{4}$ $⟶\phi_{5}$
First torsional	$\theta_{1}$	$\theta_{1}$ $⟶\theta_{2}$ $⟶\theta_{3}$ $⟶\theta_{4}$ $⟶\theta_{5}$	$\phi_{1}$ $⟶\phi_{2}$ $⟶\phi_{3}$ $⟶\phi_{4}$ $⟶\phi_{5}$
Second torsional	$\theta_{1}$ $\;\mathrm{or}\;\theta_{5}$	$\theta_{1}$ $⟶\theta_{2}$ $⟶\theta_{3}$ $\theta_{5}⟶\theta_{4}$ $⟶\theta_{3}$	$\phi_{1}$ $⟶\phi_{2}$ $⟶\phi_{3}$ $\phi_{1}⟶\phi_{2}$ $⟶\phi_{3}$
Second bending	$\theta_{1}$	$\theta_{1}$ $⟶\theta_{2}$ $⟶\theta_{3}$ $⟶\theta_{4}$ $⟶\theta_{5}$	$\phi_{1}$ $⟶\phi_{2}$ $⟶\phi_{3}$ $⟶\phi_{4}$ $⟶\phi_{5}$
Third bending	$\theta_{5}$	$\theta_{5}$ $⟶\theta_{4}$ $⟶\theta_{3}$ $⟶\theta_{2}$ $⟶\theta_{1}$	$\phi_{1}$ $⟶\phi_{2}$ $⟶\phi_{3}$ $⟶\phi_{4}$ $⟶\phi_{5}$

**Table 3:** Representative second-order curve-fitting polynomial functions.

System Parameter	Representative Curve-Fitting Polynomial Function
Viscous damping coefficient	$\Gamma_{\bar{c}}\left(\phi \right)=0.0002\phi^{2}-0.036\phi +1.59$
Stiffness coefficient	$\Gamma_{\bar{k}}\left(\phi \right)=0.0001\phi^{2}-0.018\phi +0.79$

**Table 4:** Relative error between measured and predicted parameters.

System Parameter	Mode	Relative Error (%)	
		Direct Function	Indirect Function
Viscous damping coefficient	First bending	0.078	98.64
	First torsional	0.15	4.44
	Second bending	20.83	19.14
	Third bending	11.38	22.57
	Average	8.11	36.20
Stiffness coefficient	First bending	1.65	38.75
	First torsional	0.89	54.51
	Second bending	11.22	26.85
	Third bending	21.21	79.35
	Average	8.74	49.87

## Data Availability

The data presented in this study are available on request from the corresponding author.
